# Association Between Radioactive Iodine Treatment for Pediatric and Young Adulthood Differentiated Thyroid Cancer and Risk of Second Primary Malignancies

**DOI:** 10.1200/JCO.21.01841

**Published:** 2022-01-19

**Authors:** Elisa Pasqual, Sara Schonfeld, Lindsay M. Morton, Daphnée Villoing, Choonsik Lee, Amy Berrington de Gonzalez, Cari M. Kitahara

**Affiliations:** ^1^ Division of Cancer Epidemiology and Genetics, National Cancer Institute, Rockville, MD; ^2^ Institut de Cancérologie de l’Ouest, Saint-Herblain, France

## Abstract

**PURPOSE:**

Since the 1980s, both the incidence of differentiated thyroid cancer (DTC) and use of radioactive iodine (RAI) treatment increased markedly. RAI has been associated with an increased risk of leukemia, but risks of second solid malignancies remain unclear. We aimed to quantify risks of second malignancies associated with RAI treatment for DTC in children and young adults, who are more susceptible than older adults to the late effects of radiation.

**METHODS:**

Using nine US SEER cancer registries (1975-2017), we estimated relative risks (RRs) for solid and hematologic malignancies associated with RAI (yes *v* no or unknown) using Poisson regression among ≥ 5- and ≥ 2-year survivors of nonmetastatic DTC diagnosed before age 45 years, respectively.

**RESULTS:**

Among 27,050 ≥ 5-year survivors (median follow-up = 15 years), RAI treatment (45%) was associated with increased risk of solid malignancies (RR = 1.23; 95% CI, 1.11 to 1.37). Risks were increased for uterine cancer (RR = 1.55; 95% CI, 1.03 to 2.32) and nonsignificantly for cancers of the salivary gland (RR = 2.15; 95% CI, 0.91 to 5.08), stomach (RR = 1.61; 95% CI, 0.70 to 3.69), lung (RR = 1.42; 95% CI, 0.97 to 2.08), and female breast (RR = 1.18; 95% CI, 0.99 to 1.40). Risks of total solid and female breast cancer, the most common cancer type, were highest among ≥ 20-year DTC survivors (RR_solid_ = 1.47; 95% CI, 1.24 to 1.74; RR_breast_ = 1.46; 95% CI, 1.10 to 1.95). Among 32,171 ≥ 2-year survivors, RAI was associated with increased risk of hematologic malignancies (RR = 1.51; 95% CI, 1.08 to 2.01), including leukemia (RR = 1.92; 95% CI, 1.04 to 3.56). We estimated that 6% of solid and 14% of hematologic malignancies in pediatric and young adult DTC survivors may be attributable to RAI.

**CONCLUSION:**

In addition to leukemia, RAI treatment for childhood and young-adulthood DTC was associated with increased risks of several solid cancers, particularly more than 20 years after exposure, supporting the need for long-term surveillance of these patients.

## INTRODUCTION

The incidence of differentiated thyroid cancer (DTC) increased by an average of 3.6% per year between the mid-1970s and mid-2010s (from 4.56 to 14.42 per 100,000 person-years) in the United States.^1,2^ With 15,445 estimated new cases in 2020, thyroid cancer ranks as the second most common cancer in people younger than age 45 years in the United States.^3^ Use of radioactive iodine (RAI) for DTC treatment also increased through 2009, especially in young patients.^4-9^ However, in addition to providing limited to no survival benefit for localized DTC, RAI is associated with increased risks of short-term and long-term adverse outcomes,^10-14^ including secondary primary malignancies (SPMs), such as leukemia.^15-22^ In response to this evidence, the 2009 and 2015 American Thyroid Association guidelines committee issued recommendations against RAI therapy for low-risk DTCs < 1 cm and in support of lower levels of RAI administered activity for larger tumors.^23,24^ A similar approach was suggested in the pediatric guidelines, although without explicit discouragement of RAI treatment for low-risk DTCs, and the subject remains controversial.^25-27^

**CONTEXT**

**Key Objective**
We investigated whether radioactive iodine (RAI) therapy for childhood or young adulthood thyroid cancer is associated with increased risk of second primary malignancies. Although younger individuals are more susceptible to the carcinogenic effects of radiation, the few previous studies on young patients with thyroid cancer receiving RAI therapy have been small or included insufficient follow-up time for second malignancies.
**Knowledge Generated**
Among 2-year and 5-year survivors of pediatric or young-adulthood differentiated thyroid cancer identified from nine US cancer registries (1975-2017), RAI therapy was associated with 51% and 23% increased risks of total hematologic (including leukemia) and solid malignancies, respectively. Risks of total solid cancer and female breast cancer, the most common solid cancer type, were particularly elevated after more than 20 years of follow-up.
**Relevance**
These findings may help to guide treatment decision making in younger patients with thyroid cancer to ensure that the benefits of RAI therapy outweigh the risks.

Children, adolescents, and young adults are particularly vulnerable to radiation-related secondary effects as a result of their increased tissue susceptibility and longer life expectancy.^28^ Of the few studies focused on risk of SPM after RAI for DTC in young patients, an increased risk for leukemia has been reported, specifically myelodysplastic syndrome (MDS) and acute myeloid leukemia (AML).^6,7,22^ The results for solid malignancies have been less consistent, suggesting increased risks of salivary gland and kidney cancers^6,7,10,18,29,30^; however, they were limited by short follow-up, small sample size, and lack of consideration of a minimal 5-year latency period between radiation exposure and solid malignancy development.^31,32^

Here, we aimed to estimate risk of SPM associated with RAI treatment for DTC in childhood and young adulthood (age < 45 years) using the US SEER cancer registry database (1975-2017). In addition to confirming the more well-established association of RAI treatment and leukemia risk, the inclusion of nearly 10 more years of follow-up compared with earlier SEER-based studies on this topic^6,7^ allowed for more precise assessment of solid malignancy risks.

## METHODS

### Cohort Definition

Individuals diagnosed with a first primary DTC (papillary or follicular carcinoma, Data Supplement, online only) before age 45 years between 1975 and 2017 were identified within nine SEER registries (San Francisco-Oakland, Connecticut, Detroit, Iowa, Hawaii, New Mexico, Seattle, Utah, and Atlanta),^33^ covering approximately 9% of the US population. Cases with distant metastasis at diagnosis (on the basis of Historic Stage A variable) were excluded. DTCs were further classified according to size and lymph node involvement, on the basis of the TNM definitions of the American Joint Committee on Cancer (AJCC), 7th and 8th editions.^34,35^ Age 45 years was used as the cutoff for young-adulthood DTC for consistency with AJCC staging definitions^34^ and previous studies.^6,22^ Sensitivity analyses restricted DTC cases to those diagnosed before age 40 years.^36^

Follow-up for analyses of hematologic and solid malignancies started, respectively, 2 or 5 years after DTC diagnosis, accounting for the minimal latency period for radiation-induced carcinogenesis.^31^ This exclusion also reduced the potential for surveillance bias, as the likelihood of incidental cancer detection is highest immediately following DTC diagnosis.^37^ Follow-up ended at SPM diagnosis, death, last follow-up, or December 31, 2017, whichever occurred earliest.

### Exposure and Outcome Definition

In SEER, radiation given as part of the first course of therapy is classified as yes or “no or unknown,” with “no or unknown” defined as the absence of evidence of radiation therapy during medical record review. RAI therapy was defined as receipt of radioisotope therapy after 1988 or “other radiation” (n = 1,116) or “radiation NOS” (n = 20), as opposed to external beam radiotherapy, before 1988. Patients whose radiation treatment was recorded as beam radiation before 1988 or “other radiation” or “radiation NOS” (n = 898) after 1988 were excluded.^6,7,21^

The outcomes of interest were second solid malignancies (excluding second thyroid cancer) and hematologic malignancies. Solid malignancies were categorized according to site (Data Supplement) and radiation absorbed dose delivered to normal tissue (using 0.1, 0.5, and 1 Gy cutpoints) for 100 mCi of RAI administered activity, as derived from the literature (Data Supplement).^38^

The classification of hematologic malignancies evolved over time. Before 2001, MDS and myeloproliferative neoplasms (MPN) were not captured in SEER.^39,40^ To evaluate the impact of these changes, we performed a secondary analysis of myeloid malignancies, including MDS and MPN (Data Supplement), using SEER-18 (2000-2017),^41^ which covers a shorter timeframe but larger US population proportion (28%).

### Statistical Methods

Using SEER-9 (or SEER-18) data, we obtained the observed and expected number of each SPM site. Expected numbers were derived from SEER cancer incidence rates, stratified on age (5-year groups), calendar period (5-year groups), sex, and race (White, Black, other). We conducted both an internal and an external risk comparison.

#### Internal comparisons (primary analysis).

Among patients with DTC, we estimated relative risks (RRs) and 95% CIs of SPMs according to receipt of RAI (yes *v* no or unknown) using multivariable Poisson regression models that were adjusted through stratification for sex, age at DTC diagnosis, and latency (time between DTC and SPM diagnoses) and were indirectly adjusted for attained age and calendar year using the log of the expected number of cases as an offset.^42^ RRs for solid and breast cancers (the most common SPM in this population) were also stratified by race, sex, age at exposure, follow-up, year of diagnosis, and DTC stage. Tests for heterogeneity were conducted using likelihood ratio tests.

Using parameters from the Poisson regression models, we estimated excess numbers of solid, breast, and hematologic malignancies attributable to RAI exposure. By dividing this number by the total number of second primary malignancies in ≥ 1-year survivors, we obtained a conservative estimate of the proportion of second cancers in all DTC survivors attributable to RAI (attributable risk), assuming no excess solid or hematologic malignancies were attributed to radiation in the first 5 and 2 year after exposure, respectively.

#### External comparisons (secondary analysis).

For consistency with earlier SEER-based studies,^6,7^ we computed standardized incidence ratios (SIRs) and 95% CIs for RAI-treated and non–RAI-treated patients. SIRs are the observed divided by expected number of cancers. Confounding was of particular concern with external comparisons, owing to the differences between patients with DTC and the general population (eg, health care access, socioeconomic status, lifestyle characteristics, and smoking status).^43-46^ To evaluate confounding by smoking, we compared SIRs for smoking-related versus non–smoking-related cancers.^47^ We also estimated the standardized mortality ratio (SMR) for chronic obstructive pulmonary disease (COPD), a smoking-related but not radiation-related outcome.^48^

#### Cumulative incidence.

We estimated cumulative incidence of second solid and hematologic malignancies by time since DTC diagnosis, accounting for competing risk of death and other cancers, that is, hematologic malignancies were treated as competing risk for solid malignancies and vice versa.

Analyses were conducted using SEER*Stat (version 8.3.8), the AMFIT module of Epicure (version 2.00.02), and STATA (stcompet function).^49^

### Ethical Approval

Ethical approval was not required for the use of anonymized publicly available data.^50^

## RESULTS

**FIG 1.**
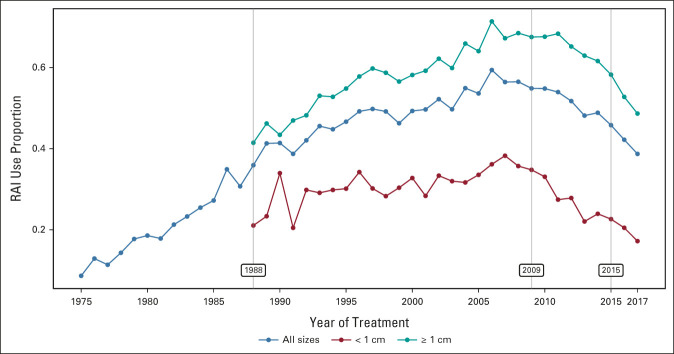
Trends in RAI therapy for nonmetastatic differentiated thyroid cancer in patients younger than 45 years in the SEER 9 (1975-2017) database, overall and by tumor size at diagnosis. 1988: change in SEER coding definitions for radiation therapy for DTC and first availability of tumor size information. 2009 and 2015: years of publication of ATA guidelines recommending against routine use of RAI for low-risk DTC. ATA, American Thyroid Association; DTC, differentiated thyroid cancer; RAI, radioactive iodine.

We identified 36,311 pediatric and young adults diagnosed with nonmetastatic DTC (accounting for 97% of all DTCs) during 1975-2017. Of these, 81% were female and 45% were treated with RAI (Table 1). RAI use was higher among males (50%) than females (44%), highest among patients younger than age 15 years (55%), and lowest in Black patients (40%). Overall, RAI use increased markedly from 9% to 55% during 1975-2009 and subsequently declined to 39% in 2017 (Fig 1). Similar trends were observed by DTC size. Maximum follow-up was 43 years.

**TABLE 1.**
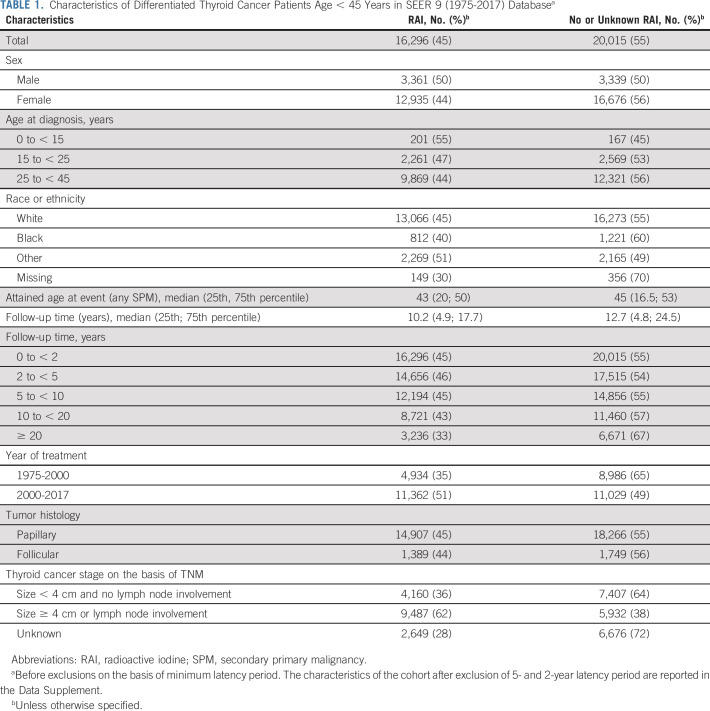
Characteristics of Differentiated Thyroid Cancer Patients Age < 45 Years in SEER 9 (1975-2017) Database^a^

### Second Solid Malignancies

**FIG 2.**
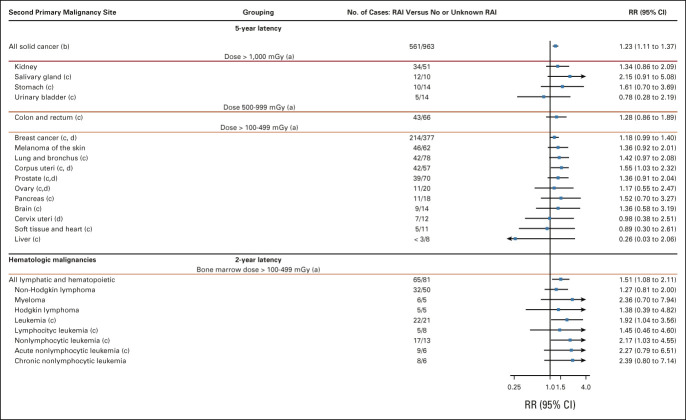
RR and 95% CI for solid and hematologic malignancies, adjusted for age at DTC diagnosis, sex, and latency. (a) Dose groups were defined according to the reported absorbed dose per administered activity in ICRP Publication 128, on the basis of an administered activity of 100 mCi in a 15-year-old patient.^38^ See the Data Supplement for details. Within each dose group, solid cancer sites are ordered according to the number of events observed in RAI-treated patients. (b) The category all solid cancer includes, in addition to the ones listed in this table, those cancer sites with < 10 events total (see the Data Supplement for details). (c) Radiosensitive organs. Those are organs for which a dose-response relationship has been described in the Japanese atomic bomb survivors' study.^64,77^ (d) Prostate risk analysis was restricted to men only, whereas ovary, breast, and corpus and cervical uterus cancer analyses were restricted to women. DTC, differentiated thyroid cancer; ICRP, International Commission on Radiological Protection; RAI, radioactive iodine; RR, relative risk.

**FIG 3.**
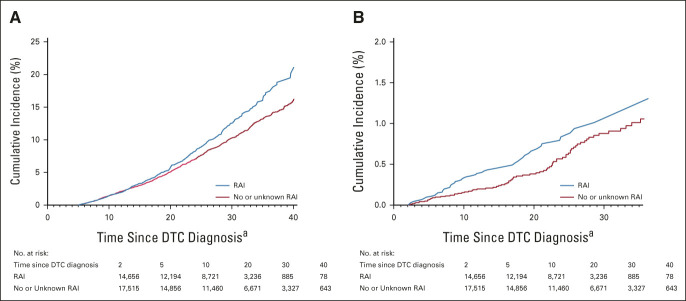
Cumulative incidence of second primary (A) solid and (B) hematologic malignancies among differentiated thyroid cancer survivors, stratified by RAI use (yes *v* none or unknown). ^a^Data were cut at 40 years (35 for hematologic malignancies) of follow-up since DTC diagnosis, because of small size of risk set after 40 years. DTC, differentiated thyroid cancer; RAI, radioactive iodine.

Among the 36,311 identified patients, 27,050 were ≥ 5-year survivors (Data Supplement), and 1,524 second solid malignancies were observed over follow-up (median = 15.6 years). RAI therapy was associated with an increased risk of solid malignancies (RR = 1.23; 95% CI, 1.11 to 1.37; Fig 2). The cumulative incidence of second solid malignancy at 20 years after DTC diagnosis was 5.6% (95% CI, 5.0 to 6.0) for RAI-treated patients and 5.0% (95% CI, 4.6 to 5.4) for those not treated with RAI, and this difference increased with subsequent follow-up (12.5% [11.3 to 13.8] and 10.2% [9.5 to 11.0], respectively, at 30 years; Fig 3). Among the highly radiation-exposed organs, RAI-associated risk was elevated for cancers of the salivary gland (RR = 2.15; 95% CI, 0.91 to 5.08), stomach (RR = 1.61; 95% CI, 0.70 to 3.69), and kidney (RR = 1.34; 95% CI, 1.14 to 2.09). A nonsignificant increased risk was observed for cancer of the colon or rectum (RR = 1.28; 95% CI, 0.86 to 1.89). For more moderately exposed organs, elevated risks were observed for cancers of the uterus (RR = 1.55; 95% CI, 1.03 to 2.32), female breast (591 cases; RR = 1.18; 95% CI, 0.99 to 1.40), and lung (RR = 1.42; 95% CI, 0.97 to 2.08). RRs were above unity for most other solid cancers except urinary bladder, cervical, and liver (Fig 2).

**FIG 4.**
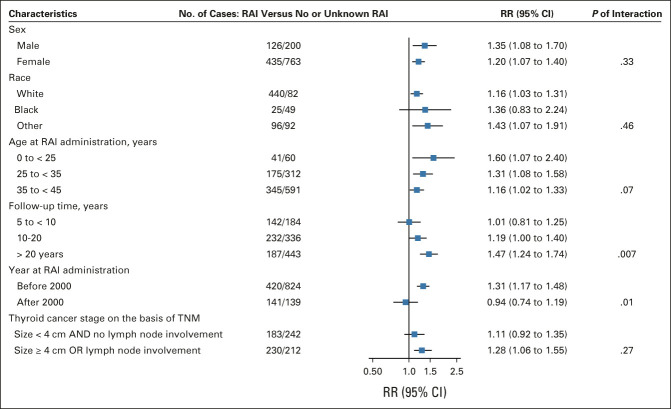
RRs and 95% CI for second solid malignancies stratified by patient and differentiated thyroid cancer characteristics. RAI, radioactive iodine; RR, relative risk.

**FIG 5.**
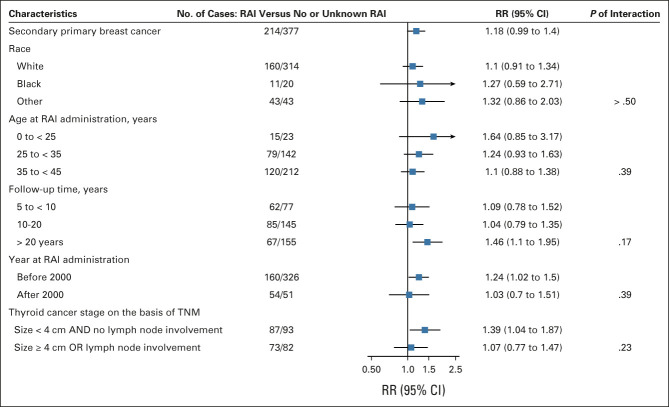
RRs and 95% CI for second female breast cancer stratified by patient and differentiated thyroid cancer characteristics. No information was available to identify premenopausal and postmenopausal breast cancer; however, 60% of breast cancer cases were diagnosed after 51 years of age, the average menopausal age in US women.^78^ RAI, radioactive iodine; RR, relative risk.

Stronger RRs for solid cancer were observed for younger versus older ages at diagnosis (*P*-trend = .07), with an RR before age 25 years of 1.60 (95% CI, 1.07 to 2.40). RRs increased with longer time since DTC diagnosis (*P*-trend = .007), with an RR after 20 years of 1.47 (95% CI, 1.24 to 1.74), and the RR was higher for those diagnosed before the year 2000 (RR = 1.31; 95% CI, 1.17 to 1.48, *P*-trend = .01; Fig 4). A similar pattern was observed for female breast cancer (Fig 5).

We estimated that 106 (95% CI, 51 to 161) excess solid cancers and 32 (95% CI, –2 to 67) excess breast cancers were attributable to RAI in this cohort, corresponding to 6% (95% CI, 3 to 9) and 5% (95% CI, 0 to 11) of all solid and breast cancers, respectively, in ≥ 1-year DTC survivors.

The SIR for second solid cancer was elevated (1.15; 95% CI, 1.06 to 1.25) among RAI-treated patients and reduced (SIR = 0.89; 95% CI, 0.83 to 0.94) among non–RAI-treated patients (Data Supplement). SIRs for lung and colon cancers were close to unity in RAI-treated patients and below one in non–RAI-treated patients; by contrast, the RRs comparing RAI-treated versus non–RAI-treated patients were elevated. The SMR for COPD was reduced in both RAI-treated (0.46; 95% CI, 0.21 to 0.88) and non–RAI-treated (0.50; 95% CI, 0.34 to 0.72) patients.

The results were similar for patients diagnosed before age 40 years (Data Supplement), with stronger RRs for lung (RR = 1.71; 95% CI, 1.04 to 2.84) and uterine (RR = 1.98; 95% CI, 1.20 to 3.28) cancer.

### Second Hematologic Malignancies

Of the 32,171 ≥ 2-year survivors (Data Supplement), 146 were diagnosed with second hematologic malignancies over follow-up (median = 13 years). RAI therapy was associated with an increased risk of leukemia (RR = 1.92; 95% CI, 1.04 to 3.56), specifically nonlymphocytic leukemia (RR = 2.17; 95% CI, 1.03 to 4.55; Fig 2). No increased risk was found for Hodgkin or non-Hodgkin lymphoma. We estimated that 22 (95% CI, 4 to 41) excess hematologic malignancies were attributable to RAI in this cohort, corresponding to an attributable risk of 14% (3 to 26). The cumulative incidence of second hematologic malignancy at 5 years after DTC diagnosis was 0.10% (95% CI, 0.06 to 0.17) for RAI-treated patients and 0.05% (0.03 to 0.10) for those not treated with RAI. At 20 years, it was 0.67% (0.49 to 0.88) and 0.37% (0.27 to 0.50), respectively (Fig 3).

#### SEER-18 (2000-2017) database.

Of the 51,902 ≥ 2-year survivors (Data Supplement), 46 second myeloid malignancies were observed over follow-up, of which 22 were MDS or AML and 24 MPN or MDS/MPN (Data Supplement). RRs were nonsignificantly increased for both MDS or AML (RR = 2.78; 95% CI, 0.90 to 8.62) and for MPN or MDS/MPN (RR = 2.77; 95% CI, 0.89 to 8.59).

## DISCUSSION

RAI is commonly used for DTC treatment, particularly in young patients.^9^ However, children and young adults are particularly susceptible to radiation-induced carcinogenesis, and they have a longer life span to develop radiation-related SPMs.^28^ In this US population–based study of survivors of pediatric and young-adult DTC, use of RAI was associated with increased risk of solid malignancies, especially after > 20 years of follow-up. This risk increased with exposure at younger ages. These findings may explain the lack of evidence of increased risk of solid malignancies in previous studies relying on shorter follow-up or focused primarily on older patients.^15,51^

Salivary gland, stomach, urinary bladder, and kidney are estimated to be highly exposed from RAI therapy for DTC (absorbed doses > 1 Gy for 100 mCi administered activity; Data Supplement) owing to the ability of these and other nearby organs and tissues to concentrate RAI.^38,52,53^ RAI therapy was associated with a two-fold risk of cancer of the salivary gland, which is highly radiosensitive,^54^ consistent with previous studies.^6,7,17,20^ Although stomach and urinary bladder are also radiosensitive,^54,55^ the kidney appears to be less so.^55^ Risks for stomach and kidney cancer were not significantly elevated, similar to previous reports.^17,18,20,30,56,57^ Based on very few cases, risk for urinary bladder cancer was not elevated. The female breast, uterus, lung, and bone marrow are also radiosensitive,^31^ and absorbed doses from RAI therapy for DTC are estimated to be between 0.1 and 0.5 Gy for 100 mCi administered activity. In our cohort, breast cancer was the most common SPM. RAI-associated risk for breast cancer was increased most markedly after 20 years of follow-up. The stronger relative risk associated with younger age at exposure is consistent with studies of other radiation-exposed populations.^32,58^

The positive findings for lung and uterine cancer, somewhat stronger in the sensitivity analysis restricted to DTC diagnosed before age 40 years, were consistent with previous studies.^17,19,59^ Studies of other radiation-exposed populations, including the Life-Span Study of Japanese atomic bomb survivors, showed a statistically significant dose-response between radiation exposure and both lung and uterine cancer, the latter being more pronounced for women exposed during puberty; however, our study included too few cases among very young patients with DTC to evaluate this.^60,61^

Our results confirmed the increased risk of hematologic malignancies following RAI treatment.^6,7,21,22,62^ It is well established that leukemia, especially AML and CML, are radiation-induced and that risk is higher for children than adults for the same unit radiation dose.^63^ No association was observed for lymphoid malignancies, which are not clearly radiation-associated.^64,65^ Statistical power was insufficient to evaluate associations for lymphoma subtypes, which are etiologically heterogeneous.^66^

Both the cumulative incidence curves and RRs stratified by follow-up were consistent in showing different exposure latency periods for hematologic and solid malignancies, with shorter-term elevated risks for hematologic malignancies and longer-term elevated risks (increasing over time) for solid malignancies. These findings are in agreement with those from other radiation-exposed populations, including the long-standing Japanese atomic bomb survivor cohorts.^31^

Important limitations of the SEER registries include underascertainment of radiation therapy and lack of detailed treatment data. SEER captures information on first course of therapy; so, any treatment received during a recurrence is not captured. Radiotherapy (including RAI) may be underascertained if not recorded in medical records reviewed by SEER; this is more likely for radiotherapy administered in outpatient settings.^67^ Underascertainment of RAI would likely bias RR estimates toward the null. As SEER does not collect information on RAI administered activity, the current study was not equipped to estimate a dose-response relationship on the basis of administered activity or patient-specific organ doses. We observed higher RRs in patients treated before 2000, potentially reflecting both longer follow-up and higher administered activity. According to previous studies, higher RAI administered activity is associated with greater risk of SPMs, with risks most evident over 100 mCi.^17-19,68-70^ To our knowledge, no previous studies of patients with DTC have evaluated these risks in relation to organ dose, the gold standard exposure measure in radiation epidemiology studies.^31^ In a recent study of patients treated for hyperthyroidism, higher organ doses were positively associated with total solid cancer and female breast cancer mortality and nonsignificant positive associations were observed for other solid cancer outcomes, including uterine cancer mortality.^71^ Although DTC therapy uses higher administered activities compared with hyperthyroidism therapy, this does not necessarily translate into higher organ doses. Correlations between administered activity and organ-specific doses depend partly on the volume of remaining thyroid tissue and its uptake of radioiodine.^52^ Finally, underascertainment of SPM because of migration outside the SEER catchment areas may have reduced the precision of RRs but was not expected to have biased these estimates.

Despite these limitations, SEER registries are population-based, avoiding potential selection biases that could arise from hospital-based studies. The large size of the cohort, long duration of follow-up, and focus on internal cohort comparisons were also key strengths of this investigation compared with previous studies,^15,20,51,72^ especially those focused on childhood and/or young adulthood DTC survivors.^6,7,10,18,22,29^ We found that the risks of second solid cancers, including breast cancer, increased with time since diagnosis. This finding is consistent with known latency periods between radiation exposure and solid cancer occurrence,^31^ and is unlikely to be explained by medical surveillance.^37^ External comparisons (SIR estimates), which were the focus of most previous SEER-based studies on this topic, are prone to confounding by cancer risk factors that differ between DTC survivors and the general population (socioeconomic status, access to health care and cancer screenings, smoking, and body mass index [BMI]).^43-46,73^ Confounding by smoking seemed to have biased the SIRs for smoking-related cancers (eg, lung, colon, ovarian, and cervical)^17,20,74^ and the SMR for COPD in the negative direction, while confounding by BMI may have biased the SIRs for obesity-related cancers (eg, corpus uteri, kidney, colon, and postmenopausal breast) in the positive direction.^73^ Since RAI therapy was not randomly assigned in this observational study, confounding of the internal comparisons (RRs) by cancer risk factors that differ for those receiving versus not receiving RAI therapy was possible. However, confounding by smoking history and BMI was unlikely as these are not factors influencing choice of RAI therapy for DTC.^23-25^ The most important factor associated with RAI therapy for DTC, apart from stage at diagnosis, is physician preference,^9,75^ which is unlikely to influence SPM risk. Long-term surveillance for SPMs is unlikely to differ by receipt of RAI therapy, as supported by a recent study that reported no difference in level of worry about SPMs for patients treated with or without RAI.^76^

We estimated that 6%, 5%, and 14% of second solid, breast, and hematologic malignancies, respectively, occurring in ≥ 1-year DTC survivors may be attributable to RAI. This is likely a conservative estimate, as the current study was not designed to estimate lifetime risks. The modest RRs together with the relative rarity of SPMs in DTC survivors means that the absolute lifetime excess risks associated with RAI are likely to be small.

Although it has been generally accepted that RAI therapy for DTC increases the leukemia risk beginning around 2-3 years after exposure, our study of pediatric and young adult patients with DTC indicates that, in the longer term, RAI increases the risk of several types of solid cancer, including breast cancer. Continued discussion and evaluation of RAI therapy in the management in DTC in pediatric and young adult patients is warranted to ensure that the benefits outweigh the risks.
